# *Trichodesmium* metaproteomes reflect the differential influence of resource availability across ocean regions

**DOI:** 10.1093/ismejo/wraf120

**Published:** 2025-06-06

**Authors:** Hanna S Anderson, Kyle R Frischkorn, Sheean T Haley, Sonya T Dyhrman

**Affiliations:** Department of Earth and Environmental Sciences, Columbia University, New York, NY 10027, United States; Lamont-Doherty Earth Observatory, Columbia University, Palisades, NY 10964, United States; Lamont-Doherty Earth Observatory, Columbia University, Palisades, NY 10964, United States; Springer Nature, Nature Portfolio, 1 New York Plaza, Suite 4600, New York, NY 10004, United States; Lamont-Doherty Earth Observatory, Columbia University, Palisades, NY 10964, United States; Department of Earth and Environmental Sciences, Columbia University, New York, NY 10027, United States; Lamont-Doherty Earth Observatory, Columbia University, Palisades, NY 10964, United States

**Keywords:** Trichodesmium, metaproteomics, diazotrophy, co-limitation

## Abstract

The diazotroph *Trichodesmium* is an important contributor to marine dinitrogen fixation, supplying nitrogen to phytoplankton in typically nitrogen-limited ocean regions. Identifying how iron and phosphorus influence *Trichodesmium* activity and biogeography is an ongoing area of study, where predicting patterns of resource stress is complicated by the uncertain bioavailability of organically complexed iron and phosphorus. Here, a comparison of 26 metaproteomes from picked *Trichodesmium* colonies identified significantly different patterns between three ocean regions: the western tropical South Pacific, the western North Atlantic, and the North Pacific Subtropical Gyre. *Trichodesmium* KEGG submodule signals differed significantly across regions, and vector fitting showed that dissolved iron, dissolved inorganic phosphorus, and temperature significantly correlated with regional metaproteome patterns. Patterns of iron and phosphorus stress marker proteins previously validated in culture studies showed significant enrichment of a phosphorus stress signal in the western North Atlantic and an iron stress signal in the North Pacific. Populations in the western tropical South Pacific appeared to modulate their proteomes in response to both dissolved iron and dissolved inorganic phosphorus bioavailability, with significant enrichment of iron and phosphorus stress marker proteins, concomitant proteome restructuring, and significant decreases in the relative abundance of the dinitrogen fixation protein, NifH. These signals recapitulate established regional patterns of resource stress on phytoplankton communities released from nitrogen stress. Evaluating community stress patterns may therefore predict resource controls on diazotroph biogeography. These data highlight how *Trichodesmium* modulates its metabolism in the field and provide an opportunity to more accurately constrain controls on *Trichodesmium* biogeography and dinitrogen fixation.

## Introduction

Nitrogen (N) limits primary production over vast swaths of the global surface tropical and subtropical oceans [1, 2]. N_2_ fixation by diazotrophs is estimated to meet roughly half of “new” N demand in marine ecosystems [3], fueling new phytoplankton primary production and making diazotrophs a keystone component of marine microbial ecosystems. Cyanobacterial diazotrophs, like members of the genus *Trichodesmium,* can thus exert profound influence over the cycling of C and N in regions where they occur [4–7]. Given their importance, ascertaining the drivers of diazotroph growth, biogeography, and N_2_ fixation is a topic of intensive study [8–22]. Of the many factors that affect diazotroph physiological ecology, iron (Fe) and phosphorus (P) are areas of focus as the N_2_-fixing enzyme nitrogenase has an Fe cofactor [23] and N_2_ fixation requires a large amount of ATP [24, 25]. Fe and P have been shown to influence diazotroph distribution and N_2_ fixation [26–30]. For instance, Fe availability has been found to influence the distribution and activity of diazotrophs in the western North Pacific [31], and the conservation of Fe stress responses across *Trichodesmium* clades suggests the universal importance of adaptation to low-Fe conditions [32]. Additionally, P has been reported to limit N_2_ fixation rates in the western North Atlantic [24, 25] and the western tropical North Pacific [33].

Recent synthesis efforts have identified the widespread occurrence of apparent resource co-limitation [1], where more than one resource was required to increase biomass in ocean surface phytoplankton community bioassays. This observation is at odds with paradigms such as Liebig’s law [34], where only a single primary resource is considered the limiting factor on biomass production [35], and complicates the identification of drivers of growth or N_2_ fixation for diazotrophs in a mixed community. It is difficult to fully resolve how resources influence a single species *in situ*, due to both the uncertain bioavailability of organic forms of limiting resources and resource competition within microbial communities. For example, *Trichodesmium* has pathways for hydrolyzing phosphate from dissolved organic P (DOP) compounds [36, 37] and acquisition pathways for particle-derived Fe have been observed [38], but the concentrations, composition, and bioavailability of these compounds are difficult to constrain. Geochemical measurements of inorganic dissolved Fe (dFe) and dissolved inorganic phosphate (DIP), therefore, do not fully represent bioavailable sources. This uncertainty has led to the study of transcripts or proteins that are more abundant under resource limitation [39], serving as species-specific markers of resource stress that can be used to survey field populations [10, 40–42].

Metaproteomic studies of marine microbial communities are expanding and offer a mechanism for linking biogeochemical and physiological patterns to environmental microbes and communities [43, 44]. As proteomic studies with model marine microbes in culture link proteins to regulation patterns, resolving the extent to which proteomes are modulated by resource supply or other factors is increasingly tractable. For example, culture studies of *Trichodesmium erythraeum* IMS101 have identified high-affinity binding proteins and enzymes that increase with Fe, P, or Fe-P co-stress [20, 21, 45–47]. Here, the term “stress” indicates a physiological response to low resource availability, distinct from limitation as stress may or may not lead to subsequent growth limitation [48]. In sum, metaproteomic studies represent a valuable tool for resolving patterns of *Trichodesmium* metabolism and identifying potential drivers of N_2_ fixation in the field. Here, *Trichodesmium* colony metaproteomes were examined to identify patterns of *Trichodesmium* physiological ecology across biogeochemically-distinctive ocean regions.

## Materials and methods

### Sample collection

*Trichodesmium* colonies were collected over seven cruise expeditions sampling either a transect (western tropical South Pacific and western North Atlantic samples) or a time-series (North Pacific samples) (Table S1) with a 130 μm mesh net hand-towed in the upper 25 m as described elsewhere [42]. Within roughly 15 minutes of collection, colonies were picked and washed 2–3 times with 0.2 μm filtered local surface water, and then collected on 5 μm 47 mm polycarbonate filters at low vacuum pressure (<170 mbar) as previously described [49]. Filters were flash frozen and stored in liquid N_2_ until protein extraction. Sampling occurred in late spring in the western North Atlantic, in late austral summer in the western tropical South Pacific, and in late summer in the North Pacific Subtropical Gyre (NPSG) near station ALOHA (Table S1). It is best to constrain sampling times due to diel variations in cell physiology and NifH protein expression [50]. Herein, metaproteome sampling times were in the morning (~7:25 a.m.–11 a.m. local time), except for some North Pacific samples which were sampled in the afternoon (11 a.m.-2 p.m. local time) due to shipboard constraints (Table S1). Samples for DIP, dFe, and either nitrate plus nitrite (N + N, North Atlantic and North Pacific samples) or NO_3_^−^ (South Pacific samples) were collected in the mixed layer and co-located with metaproteome samples with the exception of dFe data in the North Atlantic (Table S1). Although samples are referred to by region, they reflect local conditions at the time of sampling. See Supplemental Information (SI methods) for further details.

### Metaproteome analyses and data processing

Proteomic quantification was done via extraction, digestion, and liquid chromatography mass spectrometry (LC–MS) by the University of California at Davis Proteomics Core Facility (Davis, CA, United States). Briefly, polycarbonate filters with picked *Trichodesmium* were placed in 2 ml tubes with 1 ml sample extraction buffer (5% SDS, 50 mM TEAB) and heated at 95°C for 10 minutes before a 1-hour incubation with low speed shaking at room temperature. Samples were centrifuged at high speed for 20 minutes and protein concentration was measured using a bicinchoninic acid assay (Thermo Scientific). A 150 μg volume was used for S-Trap (PROTIFI) digestion before fluorescent peptide quantification (Thermo Scientific). During S-Trap digestion, proteins were reduced and alkylated, buffer concentrations were adjusted to a final concentration of 5% SDS 50 mM TEAB, 12% phosphoric acid was added at a 1:10 ratio with a final concentration of 1.2%, and S-trap buffer (100 mM TEAB in 90% MEOH) was added at a 1:7 ratio. Protein lysate S-trap buffer mixture was spun through the S-trap column and washed 3 times with S-Trap buffer. 50 mM TEAB with 6ug of trypsin (1:25 ratio) was added and the sample was incubated overnight with one addition of 50 mM TEAB and trypsin after 2 hours. Digested peptides were released by spinning at 1 minute for 3000 g with a series of solutions starting with 50 mM TEAB, then 5% formic acid, followed by 50% acetonitrile, with 0.1% formic acid. The solution was vacuum-centrifuged to near-dryness and resuspended in 2% acetonitrile (ACN), and 0.1% TFA (trifluoroacetic acid).

Nano-scale liquid chromatographic tandem mass spectrometry (nLC-MS/MS) was used to analyze tryptic peptides using an ultra-high-pressure nano-flow Easy nLC (Bruker Daltonics, Bremen, Germany). LC was done at 40°C at 400 nL min^−1^ on a reversed-phase column (50 cm × 75 μm, pulled emitter tip) with 1.9 μm C18-coated porous silica beads. Mobile phase A was done with water with 0.1% formic acid (v/v) and mobile phase B was done with 80/20/0.1% ACN/water/formic acid (v/v/vol). Peptide separation was done in a 60 min gradient (2 to 27.5% B within 45 min, increase to 40% B within 7 min, washing step at 90% B for 4 minutes, and re-equilibration).

MS was done on a hybrid trapped ion mobility spectrometry-quadrupole time of flight mass spectrometer (timsTOF Pro, Bruker Daltonics) with a modified nano-electrospray ion source (CaptiveSpray, Bruker Daltonics) operated in PASEF mode. Desolvated ions entered the vacuum region through the glass capillary and deflected into the TIMS tunnel which is electrically separated into two parts (dual TIMS) [51]. Data were acquired with a 100 ms ramp and 10 PASEF MS/MS scans per topN acquisition cycle. Low-abundance precursors with an intensity below a “target value” were repeatedly scheduled for PASEF-MS/MS scans until the summed ion count reached the target value (20 000 arbitrary units, a.u.) and MS/MS spectra were recorded from 100–1700 m/z (1 K0^−1^ of 0.7 to 1.50 V s cm^−2^, ramp time 0.85 msec). Peptide precursors were differentiated from charged background ions by applying a polygon filter to the m/z and ion mobility plane. Quadrupole isolation width was set to 2 Th for m/z under 700 and 3 Th for m/z over 700, and collision energy was increased stepwise as a function of ion mobility (20 to 59 eV).

MsFragger [52] with default parameters was used with a custom fasta reference sequence database compiled from metagenome sequences (detailed in the SI methods, Table S2) for protein identification. 115 common contaminant sequences and generated decoy sequences were appended to the original database for MSFragger. After alignment to the fasta reference sequence database (Table S2) a total of 438 025 peptides and 4477 proteins were detected across 26 samples. For protein identification, a maximum of two missing cleavages were allowed during peptide alignment, seven amino acids was the minimum required peptide sequence length, and 4600 Da was the maximum peptide mass limit; carbamidomethylation of cysteine residues was set as a fixed modification, and methionine oxidation and acetylation of protein N termini were set as variable modifications; initial maximum mass tolerances were 70 ppm for precursor and 35 ppm for fragment ions. A reversed sequence library was used to control the global false discovery rate at less than 1% for peptide spectrum matches and peptide organization into protein groups. Proteins with decoy database hits, those identified as potential contaminants, and proteins labeled by only one site modification were excluded from analysis. Label-free protein quantification was done with the IonQuant algorithm [53]. Protein clustering and normalization was done in ScaffoldLFQ (v 4.0.2). In Scaffold, normalization is done by multiplying each spectral count by the average number of spectra in all samples divided by the total spectral count in that sample. Normalization corrects for variations in the number of spectra between samples, and spectral count values reported here reflect this normalization. Proteins with identical UniProt IDs were consolidated by adding spectral counts. Twenty-two protein clusters had peptide annotations attributed to both epibiont IDs and *Trichodesmium*. Of these clusters, 12 were removed as they were determined to have redundant UniProt IDs with other protein clusters or were unidentified and could not be resolved, and six of these clusters were assigned as *Trichodesmium* as the other taxa assigned were also cyanobacteria.

Further annotation with UniProt IDs and KEGG IDs (all annotated designations are putative), consolidation, and analysis was done in RStudio (v 1.4.1106). After annotation, clustering, and data cleaning, a cutoff was applied to proteins with an average of less than 1 spectral count in every region to remove low-abundance proteins and the remaining 1848 proteins were used in subsequent analysis. Of these proteins, 1729 were annotated as *Trichodesmium* proteins (Table S3), yielding ~34% protein coverage based on the 5076 proteins encoded in the *T. erythraeum* IMS101 genome (JGI IMG, project ID Gp0000330). This coverage is in a similar range to previous field work (1590 proteins in Held et al. [50]) as well as previous culture work with *T. erythraeum* IMS101 (1933 proteins in Frischkorn et al. [45]; 1908 proteins in Walworth et al. [21]). The 119 proteins remaining included those from bacteria (Table S3) known to be associated with *Trichodesmium* colonies [54–56]. The mass spectrometry proteomics data have been deposited to the ProteomeXchange Consortium via the PRIDE [57] partner repository with the dataset identifier PXD057942 and doi: 10.6019/PXD057942. The metagenome reference sequence database fasta file is available on Zenodo (https://doi.org/10.5281/zenodo.14187345).

### Curation of protein stress markers

Proteins selected for the Fe and P stress marker sets were up-regulated under Fe or P deplete experimental treatments relative to replete controls in prior culture studies with *T. erythraeum* IMS101 (Table S4). The P marker proteins PhnM, PhnD, PhoX, PhoA, SphX, GDPD, and SqdB (Table S4) were shown to increase under P stress [21, 45]. Several of these P stress marker proteins are near a putative pho box [37, 58], and therefore co-regulation of these protein signals likely occurs [45]. SphX and PstS are both phosphate binding proteins, but only SphX is used in the P stress marker set as it has been shown to increase under P stress whereas PstS has not [21]. The proteins IsiA, IdiA, Fld1, and FbaA (Table S4) have been shown to increase under Fe stress in prior culture studies with *T. erythraeum* IMS101 [46] and are used herein as markers of Fe stress. Proteins that were not detected in all three regions (GDPD and PhoA) were removed from all stress marker signal analyses.

To calculate Fe and P stress marker signals, the average spectral count for each marker protein in a region was calculated and divided by the average spectral count across all regions (Table S3). This normalization equalized the relative contribution of individual proteins to the set as a whole, thereby avoiding bias caused by intrinsic differences in the spectral counts. These values for each protein were plotted as the P and Fe stress signals for each region. Statistical comparisons of relative enrichment of Fe and P stress signals were made using Kolmogorov–Smirnov tests to examine the null hypothesis that the spectral counts of each protein set in a region did not deviate significantly from the spectral counts of that set in another region with *P* < .05 representing a significant result, an approach described in prior studies [49, 59]. Analysis of the potential Fe-P co-stress marker signal was done in the same way, where 18 potential Fe-P co-stress protein markers (Table S5) proposed from culture work with *T. erythreaum* IMS101 [21] were treated as a marker set and statistically tested between regions. A similar method was used to evaluate the average P stress and Fe stress marker signal by sample instead of by region (Table S6). To calculate average P stress and Fe stress marker signals by sample, each marker protein was normalized by the mean spectral count of that protein across all samples. Then, these values for all P or Fe marker proteins were averaged for each sample by marker set. These average P and Fe stress marker signals were plotted against each other and overlaid by signals for PhoX, PhoA, and NifH. Overlaid signals were first normalized by the mean spectral count of that protein across all samples, then divided by the maximum value of that protein across all samples in order to uniformly scale each overlaid signal.

### Data analysis

The heatmap was generated using pheatmap [60] in R. Spectral count averages were taken across all samples in a region, and these averages were summed by their assigned KEGG submodule. The heatmap display represents the log-transformed sum of spectra assigned to respective KEGG submodules as a percentage of the total KEGG-identified spectra. Differences between individual proteins and geochemistry across regions were examined using Kruskal-Wallis hypothesis testing and post-hoc pairwise Wilcoxon (PW) testing (*P* < .05, *P* adjust method Benjamini-Hochberg) in R. Differences in stress signal metrics (including P stress, Fe stress, and putative Fe-P co-stress marker sets) were determined by Kolmogorov–Smirnov (KS) testing in R. Further details are available in the SI methods. Code for figures, intermediate data products, and statistical testing are available at https://github.com/hannaand026/Tricho_metaproteome_Anderson_2025.

## Results and discussion

### Core and unique *Trichodesmium* proteins across regions

**Figure 1:**
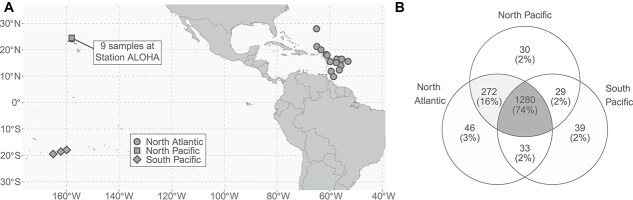
Spatial distribution of *Trichodesmium* metaproteome sampling locations and unique proteins by region. (A) Map of *Trichodesmium* samples with shape indicating the ocean regions (western North Atlantic, western tropical South Pacific, and North Pacific subtropical gyre). (B) Venn diagram of 1729 *Trichodesmium* proteins that are either shared or uniquely detected among regions based on UniProt ID.

*Trichodesmium* colonies were isolated from the western North Atlantic (herein referred to regionally as the North Atlantic) in late spring, the western tropical South Pacific (herein referred to regionally as the South Pacific) in late austral summer, and the NPSG near station ALOHA (herein referred to as the North Pacific) in late summer (Fig. 1A, Table S1), yielding 26 total samples used to identify proteins across regions. Peptides were analyzed by LC–MS and identified via mapping peptides to a custom metagenome reference database of translated protein sequences derived from metagenome assemblies of sequences from *Trichodesmium* colonies originating from each of the three sampling regions (Table S2). A total of 1848 proteins were detected of which 1729 were identified as *Trichodesmium* proteins (Table S3) and were used for subsequent analyses, whereas the remaining 119 proteins were identified as originating from an epibiont, a distinct and diverse assemblage of largely heterotrophic bacteria that associates closely with *Trichodesmium* colonies [56, 61, 62]. Most of these epibiont proteins have low spectral counts and were only detected in certain samples (Table S3), which precluded a comprehensive analysis. However, some functions are consistent with the importance of Fe and P in colony physiological ecology [7] such as a putative phosphate ABC transporter (Uniprot ID W8SRP8) and a putative ABC iron transport binding protein (Uniprot ID A8LPQ) belonging to Rhodobacterales (Table S3), lending protein expression support to previous metagenomic observations [55]. Most (74%, 1280 proteins) *Trichodesmium* proteins were detected in all three regions (Fig. 1B). For instance, the Fe-containing nitrogenase protein subunit NifH (Tery_4136) was detected in this intersection of 1280 proteins and in every sample in this study (Table S3). This NifH distribution indicates that all samples included N_2_-fixing species of *Trichodesmium*, although species that lack this ability [63] may also be present.

There were similarities in the consistent detection of abundant *Trichodesmium* proteins from different regions here (Fig. 1B) and previous observations from Station ALOHA in the North Pacific and transect cruises in the North Atlantic [41]. Of the 50 KEGG-identified proteins with the highest average spectral counts, 14 KEGG orthology IDs were shared between this and a previous study [41]. These shared high-count proteins included photosystem antennae protein CpcA (K02284), ATP synthase subunits (K02111, K02112, K02115), pentose phosphate pathway proteins (K00033, K00615), glycolysis proteins (K00927, K01810), and NifH (K02588). Despite methodological differences including sampling time and data analysis choices which can influence proteomic data outputs [43, 64], the consistent detection of these proteins across samples from different studies and locations indicates a common physiological investment of cellular resources in light-capture, carbon metabolism, ATP generation, and N_2_ fixation across *Trichodesmium* field populations from multiple environments.

In addition to high-count proteins, other proteins were consistently identified in all samples from both this and other *Trichodesmium* metaproteomes [41], including arsenite-activated ATPase ArsA (K01551) and components of a urea ABC transporter (Table S3). ArsA is part of the arsenate detoxification system that diazotrophs [65] and other cyanobacteria use to detoxify arsenate [66]. Inorganic arsenic has a nutrient-like distribution, suggesting biological cycling, with potential sources including atmospheric deposition [67]. As such, detection of arsenate detoxification transcripts or proteins is widespread in marine cyanobacterial datasets from oligotrophic systems [41, 65, 66, 68] such as those sampled in this study. ArsA expression has been reported in *Trichodesmium* metatranscriptomes from the western South Pacific [68] and widespread identification of this protein in South Pacific, North Atlantic, and North Pacific samples in this study suggests *Trichodesmium* arsenate detoxification may be broadly occurring in these oligotrophic systems. Components of a urea ABC transporter (e.g. UrtA; urea transport system substrate-binding protein) were also identified in both studies [41]. Transport and metabolism of exogenous urea may help *Trichodesmium* meet its N demand, augmenting N_2_ fixation.

Each region examined here had a relatively small percentage (2–3%) of uniquely detected proteins (Fig. 1B). This unique protein set contained two putative natural product-related proteins; CurB (K15337), a curacin-producing protein [69] in North Atlantic samples, and pesticin, a protein with lysozyme-like activity [70] in North Pacific samples (Table S3). *Trichodesmium* epibiotic bacteria, largely heterotrophs, differ significantly by ocean region and colony morphology [56], and the presence of natural product-related proteins may be related to interactions with epibiotic bacteria, or other aspects of *Trichodesmium* physiological ecology. In sum, the *Trichodesmium* metaproteomes sampled herein reflect both the presence of core functions and of regionally-distinct proteins, which may represent either local characteristics of *Trichodesmium* communities or changes in the physiology of these communities driven by geochemistry, community interactions, or other factors.

### Significant differences in protein relative abundance between regions

**Figure 2:**
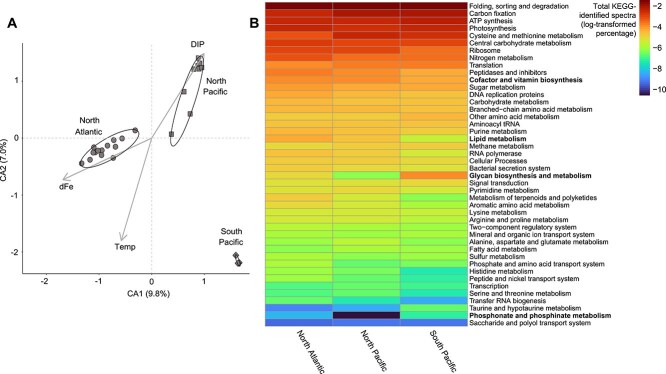
Differences in *Trichodesmium* protein spectral counts across regions. (A) Correspondence analysis of normalized spectral counts of all *Trichodesmium* proteins from all samples (PERMANOVA: *P* = 1e-4, *F* = 16.9). The explanatory variables included were determined to be significant based on vector analysis. DIP and dFe values are co-located with metaproteome samples except for western North Atlantic dFe values, which were calculated as an average of past cruise data (Table S1; see Rouco et al. [42]). (B) Heatmap of the percentage of KEGG-identified spectral counts by KEGG submodule (Table S7). Percentages are log-transformed. Samples were averaged across regions and KEGG submodule allocations were taken as a percentage of the total KEGG-identified protein spectral counts. White boxes signify that proteins from this submodule were not detected in this region. KEGG submodules in bold are examples that vary by region and are discussed in the text.

To identify potential differences in *Trichodesmium* physiology by region, metaproteomes were visualized by correspondence analysis (CA) (Fig. 2A). Although the first two dimensions of the CA analysis explain only 9.8% and 7.0% of the variance (Fig. 2A), indicating the presence of other explanatory dimensions in this dataset, metaproteome spectral counts clustered by region and were significantly different from each other (PERMANOVA, *P* = 1e-4, *F* = 16.9). These regional differences were not driven by presence-absence patterns of proteins unique to regions (Fig. S1) and likely reflect distinct environmental responses within *Trichodesmium* populations. The environmental variables (dFe, DIP, and temperature) tested for significance with vector fitting were all significant (DIP, *P* = 1e-4, *R*^2^ = 0.80; dFe, *P* = 1e-4, *R*^2^ = 0.84; temperature, *P* = 1e-4, *R*^2^ = 0.89), indicating that temperature and resource availability may influence the significant differences in *Trichodesmium* metaproteome composition across regions (Fig. 2A).

To resolve how metabolic functions varied between regions, average spectral counts of the KEGG-assigned spectra within regions were analyzed across KEGG submodules (Fig. 2B, Table S7). Percentages of total KEGG-assigned spectra showed broad consistencies in certain KEGG submodules between regions, with a largely uniform metabolic investment in core functions such as carbon fixation and photosynthesis (Fig. 2B). Other KEGG submodules such as Cofactor and Vitamin Biosynthesis, Glycan Biosynthesis and Metabolism, Phosphonate and Phosphinate Metabolism, and Lipid Metabolism varied in signal across regions (Fig. 2B), highlighting potential metabolic alterations due to abiotic or biotic factors. For example, the Cofactor and Vitamin Biosynthesis submodule increased in North Atlantic samples (Fig. 2B), driven in part by significant increases in HemN in the North Atlantic relative to the North Pacific (pairwise Wilcoxon (PW), *P* = .01; Table S3) and the South Pacific, where HemN was not detected. HemN is involved in the synthesis of the organic Fe complex protoheme (heme *b*) (C00032), whose biosynthesis has been suggested as an adaptive strategy to modulate Fe homeostasis in diazotrophs with a high Fe demand [71]. In fact, reduced dFe conditions have been linked to decreased intracellular heme *b* in marine phytoplankton [72], and the ratio of heme b:POC may be an indicator of Fe stress in phytoplankton communities [73]. A nearly complete pathway for protoheme biosynthesis (HemE, H, C, N, and L, Table S3) was detected in the North Atlantic, indicating *Trichodesmium* may increase protoheme biosynthesis in environments which receive a high flux of Fe-rich dust [74] and where Fe concentrations are typically higher than regions like the South Pacific or the North Pacific as observed here [10, 75]. Additionally, a putative ferritin (Tery_4282) (Table S3), an Fe storage protein shown to be enriched in high-Fe cultures of *T. erythraeum* IMS101 [46], was detected with significantly higher relative abundance in the North Atlantic than the North Pacific (PW, *P* = 8.8e-5), increasing evidence of possible Fe storage in this relatively Fe-rich region. Another KEGG submodule with regional differences was the Glycan Metabolism submodule, which increased in the South Pacific samples (Fig. 2B). This pattern was in part driven by an O-GlcNAc transferase (OGT) (Table S3). OGT is involved in the post-translational modification of serine or threonine residues. *Synechococcus elongatus* PCC7942 OGT mutants have higher internal concentrations of free DIP [76], hinting at a potential role in phosphate homeostasis, although this mutant was associated with additional phenotypic changes [76]. An increase in relative abundance of OGT in South Pacific samples may be related to *Trichodesmium* P physiology in this low DIP region (Table S1), or other factors. The Phosphonate and Phosphinate Metabolism KEGG submodule increased in the North Atlantic and South Pacific relative to the North Pacific (Fig. 2B), driven by increases in the relative abundance of proteins involved in the synthesis of a C-P lyase complex for the metabolism of P from phosphonates (e.g. PhnM, Table S3) [36], suggesting the use of organic P substrates by *Trichodesmium* populations in these low DIP regions (Table S1). Although these KEGG submodule patterns can be traced to potential abiotic dFe and DIP variations between regions, biotic factors, such as the interactions between epibiotic heterotrophic bacteria [55, 61, 77–79] and other organisms [80, 81] associated with *Trichodesmium* colonies, may also influence KEGG submodule signals. For example, polyketide biosynthesis proteins (Fig. 2B), including PksJ and PksL (Table S3), contributed to the increase in the Lipid Metabolism submodule signal in the North Atlantic. Some polyketides produced by *Trichodesmium* are cytotoxic [82, 83] and the *pks* gene cluster has been associated with biosynthesis of an antibacterial polyketide in other species [84]. As discussed above, the relative abundances of CurB (K15337) and pesticin also varied between regions (Table S3). Overall, the patterns in these submodules signal that a complex interplay of regionally-variable abiotic (such as dFe and DIP bioavailability) and biotic (such as colony-associated organisms) factors drive the physiological variability of *Trichodesmium* colonies across environments.

### Marker proteins indicate variation in P and Fe stress across populations

To evaluate how P and Fe bioavailability underpin *Trichodesmium* physiological ecology across regions, a set of proteins known to increase with either P or Fe stress in *T. erythreaum* IMS101 culture (Table S4) were examined. The differential abundance of marker proteins like SphX and IdiA has been used to examine patterns of *Trichodesmium* P and Fe resource stress, respectively [41]. Here, this approach was expanded to conservatively examine multiple P stress marker proteins (PhnM, PhnD, PhoX, SphX, and SqdB) or Fe stress marker proteins (IsiA, IdiA, Fld1, and FbaA) to create an integrated signal (referred to as the P or Fe stress signal) to compare between regions. Each marker protein used to derive the P or Fe stress signal (Table S4) has an established role in P or Fe physiology and has been confirmed to increase in relative abundance in culture studies as a function of either P or Fe stress [45, 46]. An increase in these P or Fe stress signals therefore represents an increase in the relative abundance of multiple proteins, each one associated with a physiological response to resource stress (Table S4).

**Figure 3:**
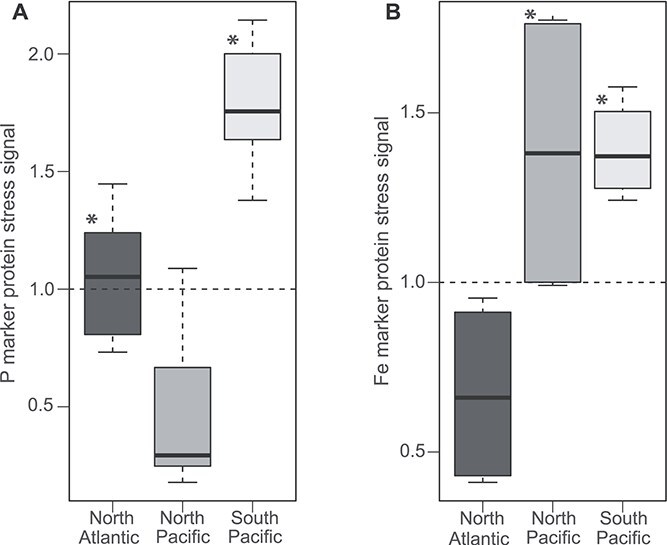
Stress signal enrichment for (A) P and (B) Fe stress signals displayed at the regional level. The P stress marker proteins included PhnM, PhnD, PhoX, SphX, SphA, and SqdB and the Fe stress proteins included IsiA, IdiA, Fld1, and FbaA (Table S4). Asterisks indicate significant differences between regions (*P <* .05) based on Kolmogorov–Smirnov testing.

The P stress signal was significantly higher in North Atlantic and South Pacific samples relative to North Pacific samples (KS, *P* < .05) (Fig. 3A). Although it can be difficult to predict patterns of P stress from DIP concentrations alone, in this case the P stress signal was highest in samples from the North Atlantic and the South Pacific, where the lowest DIP levels occurred (Table S1), and DIP concentrations did not differ significantly between South Pacific and North Atlantic samples (PW, *P* = .85). The *Trichodesmium* P stress signal in the North Atlantic samples is consistent with previous studies that identified P stress in *Trichodesmium* populations in the region using a suite of methods [24, 40, 42, 54, 85–87]. Data from the southwest Pacific has identified DIP as a key control on N_2_ fixation and on *Trichodesmium* spp. biomass in the southwest Pacific Ocean [88, 89], consistent with the metaproteome P stress signal observed here. DIP lows in austral summer and early fall are thought to drive P stress conditions in this region [89], and samples in this study taken from the western tropical South Pacific during late austral summer (February–April) had consistently low levels of DIP (8.3–16.4 nM) ([49]; Table S1). Although these samples are reflective of a specific cruise and seasonality and cannot be extrapolated beyond that, the P stress signal here supports P bioavailability as a significant driver of *Trichodesmium* physiological ecology in the North Atlantic and South Pacific samples relative to the North Pacific samples examined here.

The Fe stress signal was significantly enriched in North Pacific and South Pacific samples relative to North Atlantic samples (KS, *P* < .05) (Fig. 3B). This is consistent with lower dFe measurements in the North Pacific (0.15–0.40 nM) [74] and in the South Pacific (0.36–0.67 nM) [75] (Table S1), in contrast with typical dFe concentrations in the North Atlantic (~0.85 nM) compiled from previous studies [10, 24, 90, 91]. Moreover, dFe concentrations in the South and North Pacific samples were not significantly different from each other (PW, *P* = .06). Past environmental biomarker studies showed increases in the relative abundance of *Trichodesmium* Fe stress marker transcripts *isiA* and *idiA* in North Pacific samples compared to samples taken in the North Atlantic [10, 42], congruent with the protein signals observed here. This pattern of enhanced Fe stress in the North Pacific and South Pacific samples is also consistent with significantly higher protoheme biosynthesis and increased ferritin (associated with higher dFe) in the North Atlantic samples. In sum, the Fe stress signal suggests that *Trichodesmium* experienced elevated Fe stress in the South Pacific and North Pacific relative to the North Atlantic. These results correlate with known patterns of dFe and DIP distribution. For example, the western North Atlantic receives significant Fe-rich dust deposition compared to the other regions [92, 93], resulting in low-level DIP [94] as a consequence of increased drawdown by non-Fe limited phytoplankton [28] including *Trichodesmium* which has been shown to use dust as a source of Fe [95, 96], among other possible mechanisms.

There was significant enrichment of Fe and P stress signals in South Pacific samples relative to North Atlantic and North Pacific samples, respectively (Fig. 3). This pattern of dual-enrichment in the South Pacific is herein referred to as Fe-P co-stress, as both DIP and dFe appear to be significant drivers of *Trichodesmium* physiological ecology in this region*.* Although proteome signatures reflect short-term responses to environmental conditions [45] and may not necessarily reflect co-located geochemistry measurements, the significant differences in the Fe and P protein marker signals between regions track with the geochemistry in these regions. In the case of the South Pacific, both dFe and DIP are as low as in the North Pacific and North Atlantic, respectively (Table S1). This congruence between stress marker signals and geochemistry reinforces a scenario of Fe-P co-stress in the South Pacific relative to the other regions where stress marker signals were similarly enriched solely for either Fe or P stress signals. *Trichodesmium* is common in highly oligotrophic environments such as those sampled here, where populations are modulating their physiology to meet resource demand, and this could be reflected in joint expression of Fe or P stress markers as observed here in all samples. However, basal expression of Fe and P stress marker proteins occurs in replete cultures of *Trichodesmium* ISM101 [20, 21, 45, 46], and this complicates the interpretation and comparison of the degree of Fe and P stress, or Fe-P co-stress across field studies [40–42]. In the North Atlantic, two studies have shown that Fe and P stress markers such as IdiA and SphX are differentially modulated along inverse gradients of Fe and P, with possible Fe-P co-stress where these gradients intersect [40, 41], consistent with the patterns observed here. Taken as a whole, the South Pacific metaproteome profiles sampled here were significantly different from those from the other regions (Fig. 2A), possibly due to unique restructuring of *Trichodesmium* proteomes under Fe-P co-stress.

The significant enrichment of both Fe and P stress markers in South Pacific samples presents an opportunity to examine whether putative markers of Fe-P co-stress outlined in a previous culture experiment with *T. erythraeum* IMS101 [21] reconstruct patterns of Fe-P co-stress across field populations. Growing cultures under low dFe and DIP is challenging, and it is difficult to constrain and monitor the balance of Fe versus P stress and the magnitude of Fe-P co-stress. This makes comparing culture experiments to field populations challenging, but arguably worth trying given the importance of understanding resource controls on *Trichodesmium* physiological ecology *in situ*. Eighteen proteins which were upregulated in Fe-P co-stress relative to replete, but which did not have significant regulatory controls based on individual Fe or P stress conditions [21], were detected in metaproteomes from all three ocean regions studied here (Table S5). Treating these proteins as a putative Fe-P co-stress marker set, there was no significant enrichment in the South Pacific samples compared to the other regions (KS, *P* > .05) (Fig. S2). However, four proteins, including a fructose-1,6-bisphosphatase (Tery_0682) (PW, *P*s = .03, .03) and a mannose-1-phosphate guanylyltransferase (Tery_1856) (PW, *P*s = .03, .03), were significantly enriched in the putatively Fe-P co-stressed South Pacific samples relative to the other two regions (Table S5), and these proteins may be candidates for further study as efforts to identify Fe-P co-stress and its physiological ramifications in *Trichodesmium* field populations continue to advance.

### Functional responses to putative Fe-P co-stress

**Figure 4:**
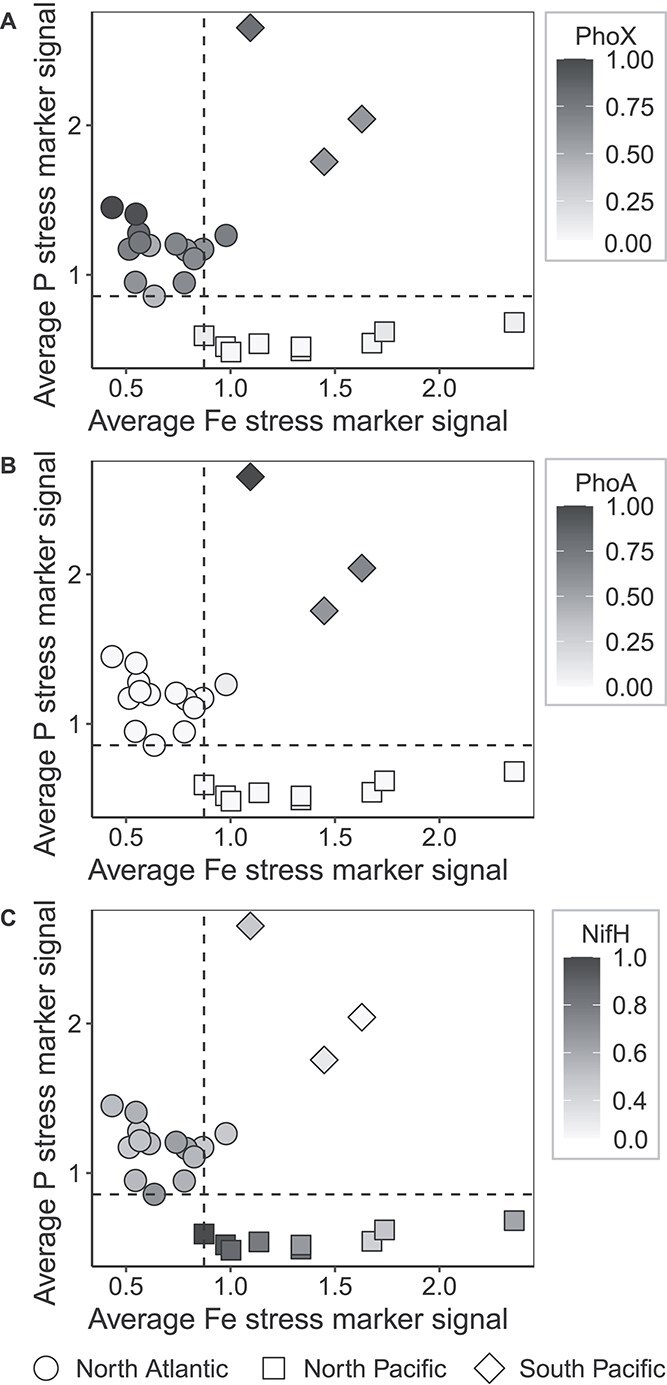
Plots of average Fe stress marker signal versus average P stress marker signal by sample (Table S6), where gray-scale overlay indicates the spectral counts of the alkaline phosphatase proteins (A) PhoX and (B) PhoA and (C) the Fe-containing subunit of nitrogenase, NifH. Overlaid spectral counts were normalized by average spectral count across all samples, then divided by the maximum value of that protein across all samples. Sample region is differentiated by shape. Horizontal and vertical lines represent minimum values of the P and Fe stress marker signals in regions experiencing single stress of P or Fe, respectively.

Co-stress in cyanobacteria can distinctively alter cell physiology, cell size, and protein expression patterns relative to single-resource stress [21, 97, 98]. Here, *Trichodesmium* proteome expression patterns in the putatively Fe-P co-stressed South Pacific samples significantly differed from the other regions with distinct KEGG submodule allocations (Fig. 2B). Fe-P co-stress may drive trade-offs different from those that occur under single-stress conditions, which has been observed in a *T. erythraeum* IMS101 culture study [20] and may in part underpin the protein expression patterns observed in the South Pacific samples. In cyanobacteria, Fe and P availability are linked by Fe co-factors in metalloenzymes involved in P metabolism, where Fe supply could constrain P bioavailability [99]. The alkaline phosphatases PhoX and PhoA are putative metalloenzymes that hydrolyze dissolved organic phosphoesters and their increased expression when phosphate drops below critical thresholds is a well-studied aspect of the P stress response in marine microbes [99–101] and in *Trichodesmium* [37, 45, 102]. These enzymes are diverse, with recent work establishing widespread atypical PhoA in cyanobacteria [103], and few have been functionally characterized [104]. PhoX typically requires Ca and Fe as co-factors at its active site [105] whereas PhoA typically requires magnesium (Mg) and zinc (Zn) [106]. These metal requirements have not been confirmed for PhoX (Tery_3845) and PhoA (Tery_3467) in *Trichodesmium*, and so putative metal co-factors must be interpreted with caution. However, growth in low DIP has been shown to increase the Zn quota in *Trichodesmium* culture studies [107], consistent with increasing Zn demand with P stress. Here, PhoX spectral counts (Table S3) were significantly lower in North Pacific samples than in North Atlantic or South Pacific samples (PW, *P*s = 7.3e-6, .01) (Fig. 4A), and PhoA increased significantly in South Pacific samples relative to North Atlantic samples (PW, *P* = 2.4e-3) and was not detected in North Pacific samples (Fig. 4B; Table S3). An increase in *phoA* transcripts in North Pacific samples relative to North Atlantic samples has been reported and it was speculated that *Trichodesmium* populations under Fe-P co-stress may favor the increased expression of *phoA* based on Fe availability [42]. The patterns observed here are consistent with a possible Fe trade-off, where populations under Fe-P co-stress favor expression of the PhoA enzyme. In other marine microbes, PhoX and PhoA have been shown to have distinct phosphoester substrate specificities [106, 108, 109]. PhoA-producing populations, such as those observed in the South Pacific, may therefore conserve Fe under Fe-P co-stress, but in doing so may alter the range of bioavailable DOP substrates. These data are consistent with Fe-P co-stress causing a trade-off between different alkaline phosphatases, where DOP bioavailability is altered as a function of Fe stress. This trade-off under Fe-P co-stress would represent a biochemically-dependent co-limitation [35] of these resources in *Trichodesmium*, where the limitation of one resource inhibits the acquisition of the other.

Several studies have identified the critical role of trace metals such as Fe or Zn in modulating alkaline phosphatase activity in low P environments [101, 110]. The expression of metalloenzymes requiring Zn is thought to underpin increases in *Trichodesmium* Zn quota during P stress [107, 111], such as the P stress observed here in the South Pacific and North Atlantic. A protein putatively identified as ZnuA (Tery_4951), a periplasmic solute-binding subunit of a high-affinity Zn ABC transporter (ZnuABC) which shares a domain (Pfam ID PF01297) with the *Escherichia coli* ZnuA [112, 113], was significantly more abundant in North Pacific and South Pacific samples relative to North Atlantic samples (PW, *P*s = 8.8e-5, 4e-3) (Table S3). In other bacteria, ZnuA is transcriptionally regulated, and is repressed when Zn is sufficient [113]. Zn is typically low in the surface ocean, but has been observed in concentrations up to three times higher in the North Atlantic surface ocean (~0.25 nM) [114] than the NPSG surface ocean (~0.07 nM) [115], which may be sufficient to support demand and repress ZnuA expression even in a low P environment. These changes in ZnuA abundance across regions are consistent with low Zn bioavailability in the North Pacific and a P stress-driven increase in Zn demand in the South Pacific, although more study is needed. This serves to reinforce possible evidence of biochemically-dependent co-limitation [35] in the South Pacific samples.

The apparent Fe-P co-stress in the South Pacific could influence rates of *Trichodesmium* N_2_ fixation, as Fe-P co-stress reduces N_2_ fixation relative to resource replete conditions in *T. erythraeum* IMS101 cultures [20]. Although *Trichodesmium* N_2_ fixation rates were not available for this data set, NifH abundance was significantly lower in South Pacific samples compared to North Atlantic and North Pacific samples (PW, *P*s = .03, .03) (Fig. 4C). This may indicate that Fe-P co-stressed populations have reduced N_2_ fixation in the South Pacific relative to other regions, consistent with the high Fe and ATP demands of N_2_ fixation and the low concentrations of both dFe and DIP relative to the samples taken in the other regions (Table S1). Diel regulation of NifH [50] could influence comparisons across regions, however most sampling, aside from one cruise in the North Pacific, was done between 7:25-11 a.m. local time, minimizing diel interference. In the South Pacific (sampled from 7:25–8:40 a.m.), the pattern of NifH relative abundance is significantly lower than North Atlantic samples (sampled from 7:25–10:20 a.m.) (PW, *P* = .03), indicating that the lower NifH signal in the South Pacific is unlikely to be driven by the sample collection time. If N_2_ fixation in the South Pacific is reduced, *Trichodesmium* may need to supplement N_2_ fixation with organic N uptake, and *Trichodesmium* populations from the same western tropical South Pacific transect sampled here took up amino acids [116], indicating organic N uptake may supplement N in Fe-P co-stressed South Pacific populations. Flavin-containing monooxygenase (FMO, Tery_3826), an enzyme capable of oxidizing trimethylamine (TMA) in marine bacteria as a source of organic N [117], was significantly more abundant in South Pacific samples, as it was only detected in one other North Pacific sample (PW, *P* = 6e-4) (Table S3). FMO has been hypothesized to scavenge organic N during a shift away from high Fe-demanding pathways including N_2_ fixation and was significantly up-regulated in Fe-P co-stressed *T. erythraeum* IMS101 cultures relative to replete controls [20]. Together, these findings are consistent with a possible shift towards organic N use and reduced N_2_ fixation in the South Pacific populations.

In addition to the significant regional pattern in NifH abundance (Fig. 4C), NifH patterns were correlated with Fe and P stress markers (Fig. S3a). All P stress marker abundances were positively correlated with each other, and NifH had a significant negative correlation with most P stress marker proteins including ones used in other studies like SphX (Fig. S3a). Similarly, Fe stress marker abundances were correlated, and several (including IdiA) had a significant negative correlation with NifH abundance (Fig. S3b). These significant inverse relationships recapitulate our observations of a decrease in NifH abundance under Fe-P co-stress. However, the opposite pattern has also been observed, where IdiA and SphX abundances were both positively correlated with NifH abundance in the North Atlantic [41]. Culture studies of *T. erythraeum* IMS101 have also shown an increase in N_2_ fixation under Fe-P co-stress relative to single Fe or P stress conditions [20]. Differences in environmental variables including the presence of organic N sources (e.g. TMA or amino acids), biological interactions with the epibiotic community which can modulate N_2_ fixation independent of Fe or P concentration [12], or the prevalence of non-diazotrophic *Trichodesmium* which still carry the Fe and P stress markers [63] could all contribute to the conflicting findings between these field datasets. It is also likely that a gradient of Fe-P co-stress exists in the environment, and the relationship between N_2_ fixation and the degree of both Fe and P stress may have nuanced effects on subsequent proteome expression patterns. For example, it has been suggested [41] that a high rate of diazotrophy may itself drive a state of local Fe-P co-stress, as the process has high demand for both Fe and P, and this could manifest differently than a scenario where low dFe and DIP environments cause reduced growth. At an intermediate level of Fe-P co-stress, NifH relative abundance may be elevated while at the same time cells have to work harder to acquire Fe and P, increasing their molecular stress signals. As resources approach scarcity, however, at a certain point Fe-P co-stress must affect the capability of the cell to fix N_2_, as N_2_ fixation has a biochemically-dependent colimitation with at least one of these resources [35]. It is possible that the South Pacific samples analyzed here are experiencing a condition of Fe-P co-stress severe enough to decrease NifH relative abundance, whereas earlier North Atlantic transect samples were experiencing an intermediate level of Fe-P co-stress where NifH relative abundance is still elevated [41]. This proposed mechanism of intermediate and severe states of Fe-P co-stress offers an explanation for the negative correlation between NifH and Fe and P stress markers reported here as well as the positive correlation seen in other field conditions [41]. An intermediate state of Fe-P co-stress supports the findings from another North Atlantic transect study [40], where increases in N_2_ fixation occurred in mid-transect samples, which displayed intermediate transcript abundances of Fe and P stress markers relative to endmember transect samples, which displayed higher relative abundances of either Fe or P marker genes as well as decreased N_2_ fixation. Culture experiments where N_2_ fixation increased under Fe-P co-stress relative to either single Fe or P stress [20] may display this intermediate pattern of Fe-P co-stress due to the constraints of experimental design and the need for a certain level of bioavailable Fe and P in order to generate experimental biomass. Defining degrees of Fe-P co-stress in field populations is challenging, but the samples examined here in the context of previous field and culture work support the idea that gradations in Fe-P co-stress could result in different physiological outcomes and protein allocation patterns. The unique proteome restructuring observed here in the South Pacific is consistent with the differential modulation of diazotroph metabolism due to the biogeochemistry of Fe, P, N, and Zn, and highlights the complex interplay of Fe, P, and N_2_ fixation in the physiological ecology of this diazotroph.

### *Trichodesmium* metaproteome patterns reflect community limitation responses

**Figure 5:**
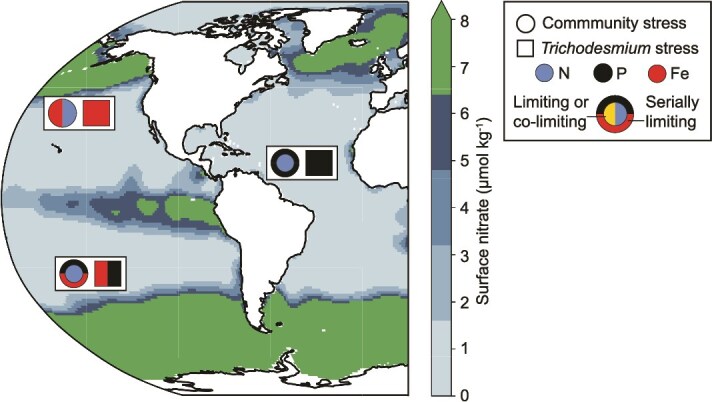
Nutrient stress patterns for *Trichodesmium* alone and the whole chlorophyll-containing community across ocean regions. *Trichodesmium* metaproteome stress patterns are displayed (squares) and compared to community stress responses [1] at locations closest to the regions sampled here (circles). Nitrogen (N) stress is indicated in blue, phosphorus (P) stress in black, and iron (Fe) stress in red. Shapes with multiple colors indicate co-stress. The outer ring indicates a serial limiting response, where adding the serially limiting nutrient enhanced growth more than adding the primary limiting nutrient alone [1]. The color scale is surface ocean nitrate (μmol kg^−1^) from the World Ocean Atlas, NOAA [118, 119].

Recent synthesis efforts of at-sea incubation studies have highlighted that serial and co-limitation of marine phytoplankton are common in the global surface ocean [1, 2]. Co-limitation (or stress, as used here) is recognized as an ecosystem-scale phenomenon, and it is thought to affect both community structure and physiology [1], driven in part by the compounding community dynamics of resource uptake and production between different community members [120]. The consequences of co-stress for *in situ* populations are still poorly understood, and reconstructing patterns of co-stress for key genera or functional groups typically requires specialized approaches such as gene frequency analyses [121] or tracking molecular expression patterns with a known response to resource availability as was done here. Previous synthesis efforts demonstrated that N was a primary limiting nutrient for phytoplankton community growth in the broader regions studied here and found that the other main limiting resources by region were Fe co-limitation (North Pacific), P serial limitation (North Atlantic), and Fe and P serial co-limitation (South Pacific) [1]. Aside from primary N limitation, these community resource limitation patterns parallel the Fe, P, and Fe-P co-stress findings determined here for *Trichodesmium* populations (Fig. 5). As such, the metaproteome analysis applied here has reinforced patterns that would have been predicted for phytoplankton released from N limitation based on a previous synthesis [1]. If this pattern holds true with further study, one could potentially use patterns of serial and co-limitation [1] to predict how resource availability controls diazotroph biogeography and N_2_ fixation. These limitation patterns highlight that keystone species like diazotrophs, with specialized ecological niches, can experience different environmental conditions relative to the larger phytoplankton community in the same location. These findings reinforce the need for molecular markers to further our understanding of the environmental conditions the microbial community experiences, as bulk samples or biogeochemical measurements cannot capture the nuanced resource dynamics, such as competition, experienced by members of the phytoplankton community.

Rising ocean temperatures are predicted to constrict *Trichodesmium* geographical distribution near the warming equator while simultaneously expanding its range overall into higher latitudes [122]. Time series data in the North Atlantic subtropical gyre have shown that oligotrophic conditions are already expanding both spatially and temporally [123] with declines in upper ocean DIP [124]. As warm, oligotrophic waters expand, diazotrophic cyanobacteria such as *Trichodesmium* may simultaneously experience more frequent environmental stress of both Fe and P and play an increasingly large role in N cycling. This may, in turn, influence the biogeochemistry and availability of other resources for the phytoplankton community— e.g. Zn. If N_2_ fixation is confirmed with further study to decrease under Fe-P co-stress, non-diazotrophs in Fe-P co-stressed regions may be critically N-limited in already low-nutrient conditions. Clarifying this complex interplay of biological demand, limiting resources, and organismal biogeography across ecosystems is vital to understanding how future ocean conditions will affect the patterns of cyanobacterial diazotrophic physiological ecology that underpin primary production.

## Supplementary Material

Fig_S1_wraf120

Fig_S2_wraf120

Fig_S3_wraf120

Supplemental_information_wraf120

Supplementary_Tables_wraf120

## Data Availability

The data supporting these findings are openly available. The mass spectrometry proteomics data have been deposited to the ProteomeXchange Consortium via the PRIDE [57] partner repository with the dataset identifier PXD057942 and doi: 10.6019/PXD057942. The reference assembly used for protein identification is available on Zenodo (https://doi.org/10.5281/zenodo.14187345). Code for figures, intermediate data products, and statistical testing are available at https://github.com/hannaand026/Tricho_metaproteome_Anderson_2025.
