# Supplementary figures and images for: Trichodesmium metaproteomes reflect the differential influence of resource availability across ocean regions
Source: ISME J. 2025 Jun 6;19(1):wraf120. doi: 10.1093/ismejo/wraf120 (PMC12206446; doi:10.1093/ismejo/wraf120)

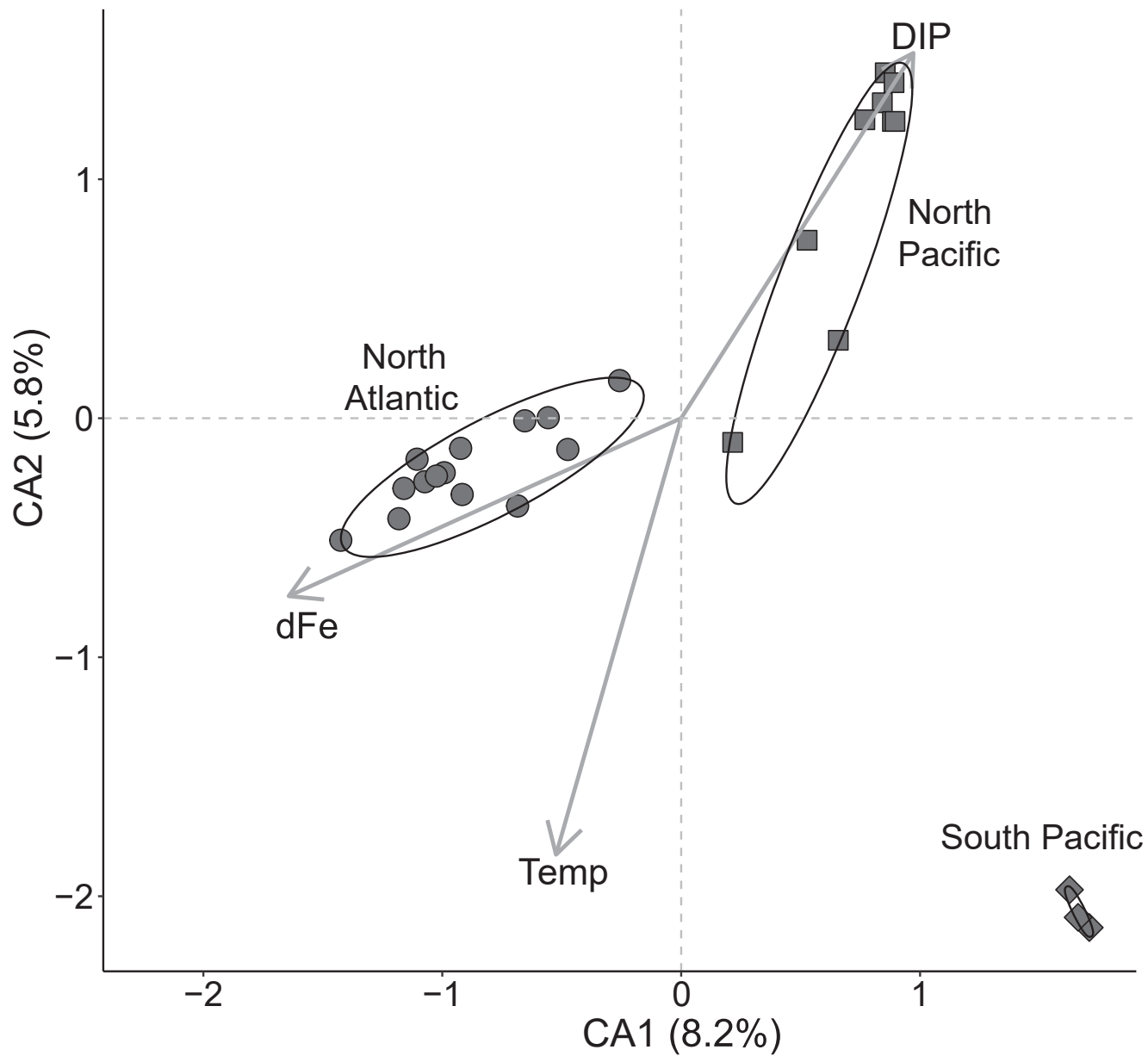

Supplement: Fig_S1_wraf120 [file fig_s1_wraf120.pdf]

Putative Fe-P co-stress marker protein signal

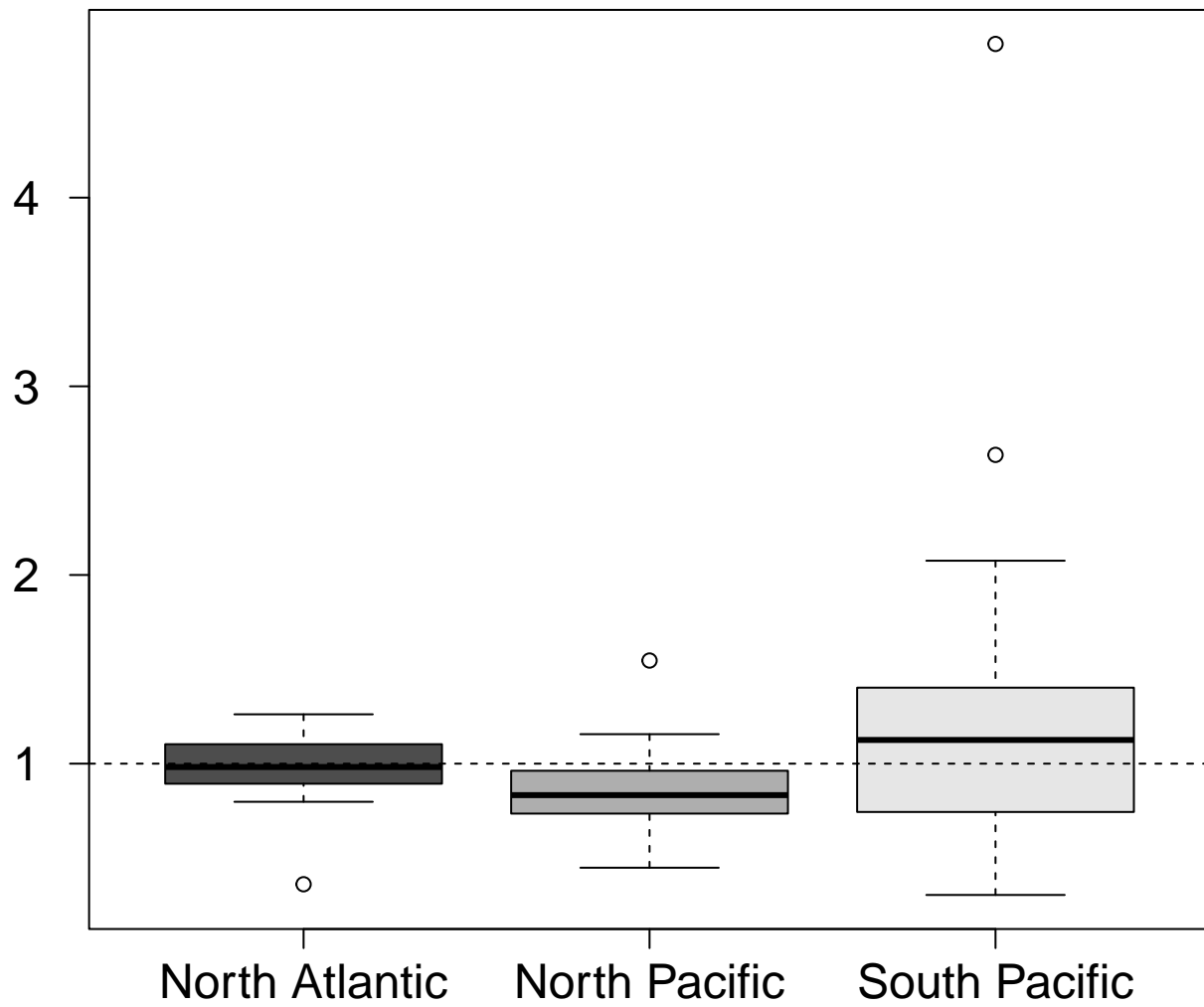

Supplement: Fig_S2_wraf120 [file fig_s2_wraf120.pdf]

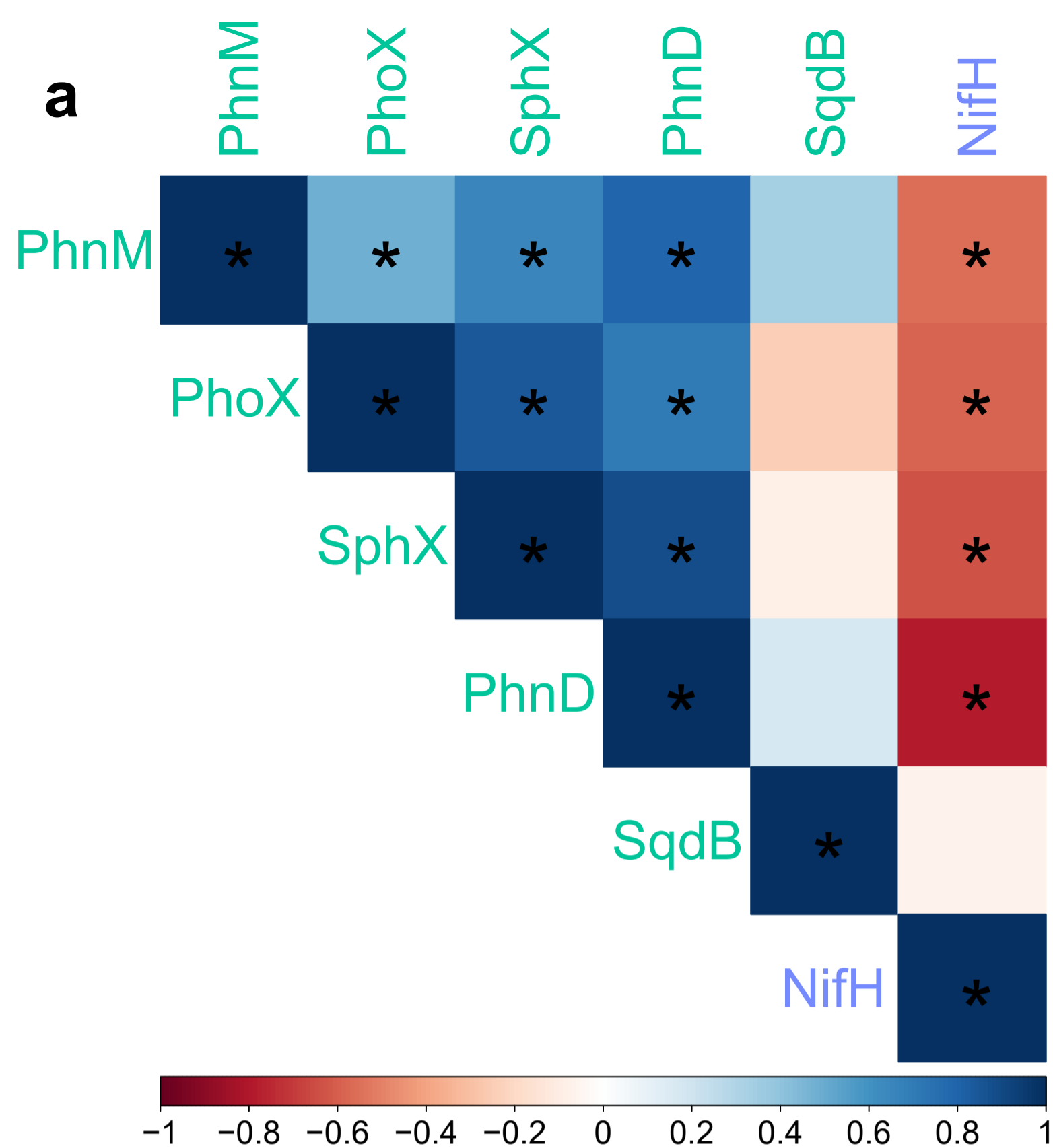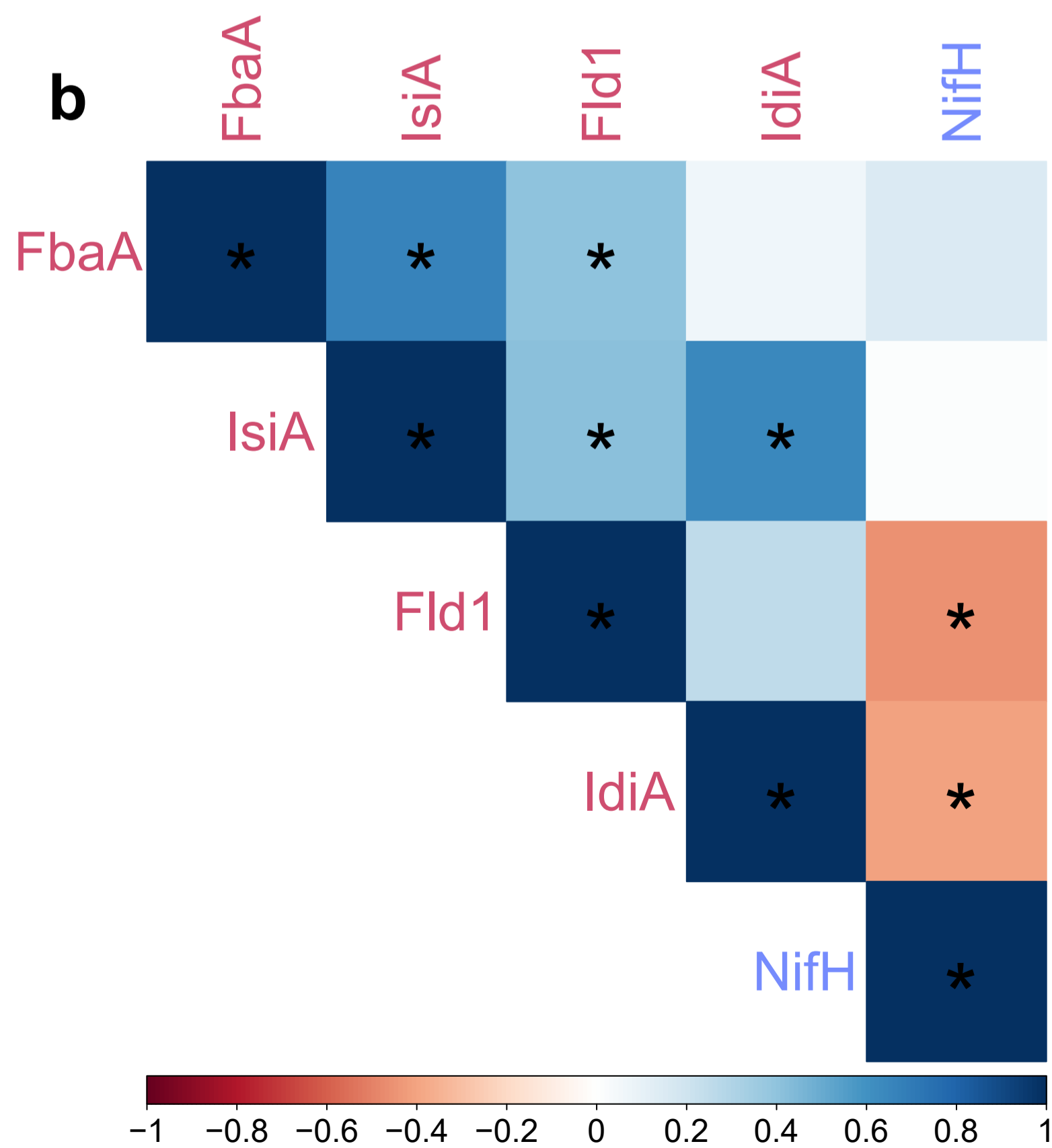

Supplement: Fig_S3_wraf120 [file fig_s3_wraf120.pdf]
