# Supplementary material for: Trichodesmium metaproteomes reflect the differential influence of resource availability across ocean regions
Source: ISME J. 2025 Jun 6;19(1):wraf120. doi: 10.1093/ismejo/wraf120 (PMC12206446; doi:10.1093/ismejo/wraf120)
Supplement: Supplemental_information_wraf120 [file supplemental_information_wraf120.docx]

**Supplemental Information**

**Supplementary Methods**

***Sample collection and environmental data processing supplement***

Environmental samples for dissolved inorganic phosphate (DIP), dFe, and NO_3_^-^ in the South Pacific (Table S1) were taken at 10 m via a titanium rosette and filtered through 0.2 μm cartridges at the same stations where *Trichodesmium* was collected (Frischkorn et al., 2018b) and DIP concentrations were determined via a flow auto-analyzer (Bonnet et al., 2018). For South Pacific samples, DIP was analyzed on board with a 2 m liquid wavelength capillary cell (LWCC) (Pulido-Villena et al., 2010) and dFe was analyzed with flow injection (Blain et al., 2008). South Pacific dFe, DIP, and NO_3_^-^ data are available in previous publications (Guieu et al., 2018; Frischkorn et al., 2018b).

Environmental samples from the North Pacific were taken near Station ALOHA (Table S1). Samples were taken at a depth of 3 m on the same date as the *Trichodesmium* collections, where the dFe was processed and measured as previously detailed (Fitzsimmons et al., 2012), and nitrate plus nitrite (N+N) and DIP concentrations were determined using a segmented flow auto-analyzer (Rii et al., 2018). DIP and N+N data is available via the Center for Microbial Oceanography: Research and Education (C-MORE), an NSF Science and Technology Center (EF-0424599) (hahana.soest.hawaii.edu/hoedylan/data, hahana.soest.hawaii.edu/hoephor/data/data.html) and dFe data is available in a previous publication (Fitzsimmons et al., 2012).

In the North Atlantic, DIP samples from cruise AE1409 were taken at a depth of ~5 m (Frischkorn et al., 2017) and analyzed using a modified MAGIC method (Rimmelin & Moutin, 2005) with a detection limit of 2.5 nM (Rouco et al, 2018), and data is available at BCO-DMO (Van Mooy & Dyhrman, 2017) and in previous publications (Frischkorn et al., 2017). N+N values from the OC471 cruise were analyzed after the protocols of the Chesapeake Bay Lab at the University of Maryland and DIP samples were also analyzed using MAGIC (Rouco et al., 2018). OC471 nutrient data is available at BCO-DMO (McGillicuddy et al., 2014) and in previous publications (Rouco et al., 2014; Olson et al., 2015a; Olson et al., 2015b). Environmental dFe data was not collected on either western North Atlantic cruise and a climatological average was determined in a previous publication (Rouco et al., 2018) based on previous samples from 1996-2004 (Sañudo-Wilhelmy et al. 2001; Wu & Boyle, 2002; Bergquist & Boyle, 2006; Chappell et al., 2012).

***Metagenome reference sequence database creation***

To create the reference database, DNA sequences from picked *Trichodesmium* samples (Table S2) were collected and processed as described elsewhere (Frischkorn et al., 2017, Frischkorn et al., 2018a; Frischkorn et al., 2018b). In brief, reads were trimmed with Sickle (https://github.com/najoshi/sickle) under default settings and assembled using IDBA-UD with default parameters (Peng et al., 2012). Assembled scaffolds were clustered into genome bins using MaxBin 2.0 with default parameters (Wu et al., 2015) and translated with the metagenomic setting of Prodigal (Hyatt et al., 2010). Taxonomic partitioning and classification of binned scaffolds was done with NCBI nr and blastp in DIAMOND (Buchfink et al., 2021) and MEGAN6 (Huson et al., 2013). Functional annotations were obtained using DIAMOND (parameters -e 0.001) against the UniRef90 database (Suzek et al., 2007) and against the Kyoto Encyclopedia of Genes and Genomes (KEGG) using the online Automatic Annotation Server (using bidirectional best-hit method, GHOSTX search program, and prokaryote representative gene set). The reference assembly used for protein identification is available on Zenodo (<https://doi.org/10.5281/zenodo.14187345>).

***Data analysis supplementary methods***

Correspondence analysis (CA) and PERMANOVA analyses were done using the vegan package (Oksanen, 2015). The CA was made using the cca function, vector fitting was done with function envfit with 9999 permutations, and PERMANOVA was run using the adonis2 function with 9999 permutations. In vector fitting, N+N was also significant (*P*=1e-4, *R*^2^=0.84), however co-located N+N data was not available for six of the nine North Atlantic samples (Table S1) and South Pacific NO_3_^-^ data was collected by a different method, and therefore this variable was not included. To ensure sample separation by region was not due to the inclusion of proteins found in only one region, a secondary CA analysis was run on only proteins shared between all regions, which showed no substantive differences in clustering or explanatory variables (Fig. S1). Colonies isolated in this study were not identified taxonomically, however there is evidence from similar transects in all 3 sampling regions that *Trichodesmium* clade I is dominant and clade III exists in lower but varying proportions throughout (Rouco et al., 2014; Rouco et al., 2016; Frischkorn et al., 2018b). As the dominant clade is the same across regions, differences in taxonomy are unlikely to be the main driver of proteome differences between regions.

In classifying metabolic modules, if a protein was not initially assigned a KEGG submodule, identification was added only if KEGG class or pathway identification could be matched with an existing KEGG submodule category (Table S7). Any submodule with fewer than three annotated proteins was not visualized (Fig. 2b), although these proteins were still counted in calculating the percentage of total KEGG-identified spectra.

Surface ocean nitrate data (μmol kg-1) was obtained from the World Ocean Atlas (NOAA) (Reagan et al., 2023; Garcia et al., 2024) and plotted in Python (v 3.10.8). Representative community stress responses by ocean region were selected (Browning & Moore, 2023) from the locations closest to those sampled in this study.

Pearson correlations of spectral counts P and Fe stress marker proteins (Table S4) and NifH spectral counts (Table S3) were done with the corrplot (Wei & Simko, 2024) and Hmisc (Harrell & Dupont, 2019) packages in R, and correlations were considered significant if *P* < 0.05. Only stress marker proteins detected in all three ocean regions studied here and used in the stress signal analysis were included in the correlation. Code for figures, intermediate data products, and statistical testing is available at https://github.com/hannaand026/Tricho_metaproteome_Anderson_2025.

**References**

Bergquist, B.A., Boyle, E.A. (2006). Dissolved iron in the tropical and subtropical Atlantic Ocean. *Global Biogeochem Cy*, 20: GB1015. Doi: 10.1029/2005GB002505.

Blain, S., Bonnet, S., and Guieu, C. (2008). Dissolved iron distribution in the tropical and subtropical South Eastern Pacific. *Biogeosciences*, 5: 269–280. Doi: 10.5194/bg-5-269-2008.

Bonnet, S., Caffin, M., Berthelot, H., Grosso, O., Benavides, M., Helias-Nunige, S., et al. (2018). In-depth characterization of diazotroph activity across the western tropical South Pacific hotspot of N_2_ fixation (OUTPACE cruise). *Biogeosciences*, 15(13): 4215–4232. Doi: 10.5194/bg-15-4215-2018.

Browning, T.J., Moore, C.M. (2023). Global analysis of ocean phytoplankton nutrient limitation reveals high prevalence of co-limitation. *Nat Commun,* 14: 5014. Doi: 10.1038/s41467-023-40774-0.

Buchfink, B., Reuter, K., Drost, H. G. (2021). Sensitive protein alignments at tree-of-life scale using DIAMOND. *Nat Methods*, 18(4), 366–368. Doi: 10.1038/s41592-021-01101-x.

Chappell, P.D., Moffett, J.W., Hynes, A.M., Webb, E.A. (2012). Molecular evidence of iron limitation and availability in the global diazotroph *Trichodesmium*. *ISME J*, 6: 1728–39. Doi: 10.1038/ismej.2012.13.

Fitzsimmons, J.N., Zhang, R., Boyle, E.A. (2013). Dissolved iron in the tropical North Atlantic Ocean. *Mar Chem*, 154: 87-99. Doi: 10.1016/j.marchem.2013.05.009.

Frischkorn, K.R., Rouco M., Van Mooy, B.A.S., Dyhrman, S.T. (2017). Epibionts dominate metabolic functional potential of *Trichodesmium* colonies from the oligotrophic ocean. *ISME J*, 11(9): 2090-2101. Doi: 10.1038/ismej.2017.74.

Frischkorn, K.R., Haley, S.T., Dyhrman, S.T. (2018a). Coordinated gene expression between *Trichodesmium* and its microbiome over day-night cycles in the North Pacific Subtropical Gyre. *ISME J*, 12(4): 997-1007. Doi: 10.1038/s41396-017-0041-5.

Frischkorn, K.R., Krupke, A., Guieu, C., Louis, J., Rouco, M., Estrada, A.E.S., et al. (2018b). *Trichodesmium* physiological ecology and phosphate reduction in the western tropical South Pacific. *Biogeosciences*, 15(19): 5761-5778. Doi: 10.5194/bg-15-5761-2018.

Garcia, HE, Bouchard, C, Cross, SL, et al. (2024). World Ocean Atlas 2023, Volume 4: Dissolved Inorganic Nutrients (phosphate, nitrate, silicate). Mishonov, A, Tech. Ed. NOAA Atlas NESDIS 92. Doi: 10.25923/39qw-7j08.

Guieu, C., Bonnet, S., Petrenko, A., Menkes, C., Chavagnac, V., Desboeufs, K., et al. (2018). Iron from a submarine source impacts the productive layer of the Western Tropical South Pacific (WTSP). *Sci Rep*, 8: 9075. Doi: 10.1038/s41598-018-27407-z.

Harrell, F. Jr., Dupont, C. (2019). Hmisc: Harrell Miscellaneous. R Package Version 4.2-0. https://CRAN.R-project.org/package=Hmisc.

Huson, D. H., El Hadidi, M., Ruscheweyh, H., Meta, S. (2013). Improved metagenome analysis using MEGAN5. Available at: http://ab.inf.uni-tuebingen.de/software/megan5/. (last access: 17 September 2018).

Hyatt, D., Chen, G-L., LoCascio, P.F., Land, M.L., Larimer, F.W., Hauser, L.J. (2010). Prodigal: prokaryotic gene recognition and translation initiation site identification. *BMC Bioinformatics*, 11: 119. Doi: 10.1186/1471-2105-11-119.

McGillicuddy, D.J., Dyhrman, S.T., Davis, C.S., Waterbury, J.B. (2014) *Trichodesmium* species in the North Atlantic from R/V Oceanus OC471-01 in the NW Atlantic: Woods Hole to Barbados from April 2011 (*Trichodesmium* project). Biological and Chemical Oceanography Data Management Office (BCO-DMO). (Version 2014-04-08) Version Date 2014-04-08. http://lod.bco-dmo.org/id/dataset/505567. Accessed May 23, 2024.

Oksanen, J. (2015). Multivariate analysis of ecological communities in R: vegan tutorial. URL: https://john-quensen.com/wp-content/uploads/2018/10/Oksanen-Jari-vegantutor.pdf.

Olson, E. M., McGillicuddy D.J., Flierl, G.R., Davis, C.S., Dyhrman, S.T., Waterbury J.B. (2015a). Mesoscale eddies and *Trichodesmium* spp. distributions in the southwestern North Atlantic. *J Geophys Res-Oceans*, 120(6): 4129–4150. Doi: 10.1002/2015JC010728.

Olson, E.M., McGillicuddy, D.J., Dyhrman, S.T., Waterbury, J.B., Davis, C.S., Solow, A.R. (2015b). The depth-distribution of nitrogen fixation by *Trichodesmium* spp. colonies in the tropical–subtropical North Atlantic. *Deep-Sea Res Pt I*, 104: 72-91. Doi: 10.1016/j.dsr.2015.06.012.

Peng, Y., Leung, H.C.M., Yiu, S.M., Chin, F.Y.L. (2012). IDBA-UD: a de novo assembler for single-cell and metagenomic sequencing data with highly uneven depth. *Bioinformatics*, 28(11): 1420–1428. Doi: [10.1093/bioinformatics/bts174](https://doi.org/10.1093/bioinformatics/bts174).

Pulido-Villena, E., Rérolle, V., Guieu, C. (2010). Transient fertilizing effect of dust in P-deficient LNLC surface ocean. *Geophys Res Lett,* 37: L01603. Doi: 10.1029/2009GL041415.

Reagan, J.R., Boyer, T.P., García, H.E., Locarnini, R.A., Baranova, O.K., Bouchard, C., et al. (2023). World Ocean Atlas 2023 (NCEI Accession 0270533). https://www.ncei.noaa.gov/archive/accession/0270533. In Reagan, J.R., Boyer, T.P., García, H.E., Locarnini, R.A., Baranova, O.K., Bouchard, C., et al. World Ocean Atlas 2023. [Annual nitrate 15m]. NOAA National Centers for Environmental Information. Dataset. https://doi.org/10.25921/va26-hv25. Accessed February 2025.

Rii, Y.M., Bidigare, R.R., Church, M.J. (2018). Differential responses of eukaryotic phytoplankton to nitrogenous nutrients in the North Pacific subtropical gyre. *Front Mar Sci,* 5: 92. Doi: 10.3389/fmars.2018.00092.

Rimmelin, P., Moutin, T. (2005). Re-examination of the MAGIC method to determine low orthophosphate concentration in seawater. *Anal Chim Acta,* 548(1-2): 174-182. Doi: 10.1016/j.aca.2005.05.071.

Rouco, M., Frischkorn, K.R., Haley, S.T., Alexander, H., Dyhrman, S.T. (2018). Transcriptional patterns identify resource controls on the diazotroph *Trichodesmium* in the Atlantic and Pacific oceans. *ISME J*, 12(6): 1486-1495. Doi: 10.1038/s41396-018-0087-z.

Rouco, M., Haley, S.T., Dyhrman, S.T. (2016). Microbial diversity within the *Trichodesmium* holobiont. *Environ Microbiol*, 18(12): 5151-5160. Doi: 10.1111/1462-2920.13513.

Rouco, M., Joy-Warren, H.J., McGillicuddy, D.J., Waterbury, J.B., Dyhrman, S.T. (2014). *Trichodesmium* sp. clade distributions in the western North Atlantic Ocean. *Limnol Oceanogr*, 59(6): 1899–1909. Doi: 10.4319/lo.2014.59.6.1899.

Sañudo-Wilhelmy, S.A., Kustka, A.B., Gobler, C.J., Hutchins, D.A., Yang, M., Lwiza, K., et al. (2001). Phosphorus limitation of nitrogen fixation by *Trichodesmium* in the central Atlantic Ocean. *Nature*, 411: 66-69. Doi: 10.1038/35075041.

Suzek, B.E., Huang, H., McGarvey, P., Mazumder, R., Wu, C.H. (2007). UniRef: comprehensive and non-redundant UniProt reference clusters. *Bioinformatics*, 23(10): 1282–1288. Doi: 10.1093/bioinformatics/btm098.

Van Mooy, B.A.S., Dyhrman, S.T. (2017). “Dissolved Phosphorus Processing by *Trichodesmium* Consortia: Quantitative Partitioning, Role of Microbial Coordination, and Impact on Nitrogen Fixation (P Processing by *Tricho*)”. Biological and Chemical Oceanography Data Management Office (BCO-DMO). Dataset version: 17 July 2017. https://www.bco-dmo.org/dataset/709621. Accessed: February 2, 2024.

Wei, T., Simko, V. (2024). R package 'corrplot': Visualization of a correlation matrix. Version 0.95. https://github.com/taiyun/corrplot.

Wu, J., Boyle, E. (2002). Iron in the Sargasso Sea: implications for the processes controlling dissolved Fe distribution in the ocean. *Global Biogeochem Cy,* 16(4): 1086. Doi: 10.1029/2001GB001453.

Wu, Y., Simmons, B.A., Singer, S.W. (2015). MaxBin 2.0: an automated binning algorithm to recover genomes from multiple metagenomic datasets. *Bioinformatics*, 32(4): 605-607. Doi: 10.1093/bioinformatics/btv638.

**Supplemental tables and figures:**

**Table S1:** Sample metadata.

**Table S2:** Sampling details and citations for metagenomes used to build the metagenome reference database.

**Table S3:** Protein spectral counts and UniProt and KEGG annotations.

**Table S4:** Marker protein list with UniProt and Tery IDs, literature citations, and putative functions.

**Table S5:** List of putative Fe-P co-stress markers (Fig. S2) which were upregulated in *T. erythraeum* IMS101 under Fe-P co-stress culture relative to replete conditions (Walworth et al., 2016) and which were detected in all three ocean regions in this study.

**Table S6:** Average P and Fe stress signals by sample (Fig. 4).

**Table S7:** KEGG submodule designations of KEGG-annotated *Trichodesmium* protein spectra used to generate heatmap (Fig. 2b). KEGG submodule designation is detailed in the SI methods.

**Figure S1:** Correspondence analysis of spectral counts of only *Trichodesmium* proteins detected in all three regions. Explanatory variables are included based on significance determined from vector analysis. DIP and dFe values are co-located with metaproteome samples except for North Atlantic dFe values, which were calculated as an average of past transect data (Table S1; see Rouco et al. (2018)).

**Figure S2:** Putative Fe-P co-stress signal metric displayed at the regional level. Putative Fe-P co-stress marker proteins (Table S5) were determined to be upregulated in *T. erythraeum* IMS101 under Fe-P co-stress relative to replete conditions, and were not regulated by either Fe or P single stress relative to replete conditions (Walworth et al, 2016). Differences between regions were not significant (KS, *P>*0.05) based on Kolmogorov-Smirnov testing.

**Figure S3:** Pearson correlations of spectral counts (Table S3) of (A) P stress marker proteins (Table S4) and NifH spectral counts and (B) Fe stress marker proteins (Table S4) and NifH spectral counts across all samples. Significant correlations (*P*<0.05) are denoted by asterisks. Only stress marker proteins detected in all three ocean regions studied here and used in the stress signal analysis are included in the correlation.
